# Investigation of the oxygen reduction kinetics and mechanism on ordered mesoporous carbon-supported Pt-based catalysts

**DOI:** 10.3906/kim-2102-68

**Published:** 2021-12-19

**Authors:** Silver GÜNEŞ, Çiğdem GÜLDÜR

**Affiliations:** 1 Graduate School of Natural and Applied Sciences, Gazi University, Ankara, Turkey; 2 Department of Chemical Engineering, Faculty of Engineering, Gazi University, Ankara, Turkey

**Keywords:** Oxygen reduction reaction, reaction mechanism, ordered mesoporous carbon, rotating disk voltammetry

## Abstract

The kinetics and mechanism of oxygen reduction reaction (ORR) on the surface of Pt and PtAgFe catalysts supported by ordered mesoporous carbon (OMC) were investigated. Metal particles were loaded by the wet impregnation-reduction method on two types of OMC, one being surface-modified by HNO_3_ and the other unmodified. Cyclic voltammetry measurements showed that Pt on modified OMC, namely Pt/OMC-M, had the highest electroactive surface area among OMC-supported catalyts with 987 cm^2^/mg. This catalyst had also a mass activity of 39.1 mA/mg_Pt_, which was the same as the carbon black supported commercial Pt/C catalyst. Rotating disk electrode (RDE) studies revealed that the net number of transferred electrons for OMC supported Pt and PtAgFe catalysts were in the range between 3 and 4, indicating a predominant 4-electron water formation route. The ORR mechanisms on Pt/OMC and PtAgFe/OMC were found to be similar with that of Pt/C; however, the associative mechanism was pronounced at a wider potential range. The modified OMC-supported catalysts showed higher oxygen reduction activities in general.

## 1. Introduction

The performance of a fuel cell heavily depends on the rate of the oxygen reduction reaction (ORR) occurring at the cathode [1]. ORR is much slower compared to the hydrogen oxidation at the anode side, therefore, limiting the overall reaction rate. As a consequence, most of the studies related to fuel cells focus on improving the kinetics of ORR through the development of efficient catalysts, supporting materials or better preparation techniques to increase the utilization and activity of already available materials [2–5]. Platinum has long been the conventional ORR catalyst despite its high cost. The kinetics of the ORR on Pt surface for both acidic and alkaline conditions was extensively studied in the literature. Damjanovic was among the first researchers to propose a detailed mechanism for ORR on Pt in alkaline media, which involved a three-step reduction starting with the adsorption of molecular oxygen on the Pt surface and followed by two consecutive electron transfer steps [6]. Studies conducted in acidic conditions showed that the reaction follows a similar path [7]. Since then, there have been many modifications to this mechanism [8,9]. In fuel cell operating conditions, it is generally accepted that the cathode side OR reaction starts with the diffusion and subsequent adsorption of an oxygen molecule on the surface of Pt, followed by electron transfer steps and desorption from the surface [10].

The ORR mechanism can basically follow two paths, depending on how the oxygen molecule is adsorbed on the Pt surface; oxygen can be adsorbed either on a single Pt atom by a single bond or on two Pt atoms by two bonds. The single-bond adsorption leads to the associative mechanism, while double-bonded adsorption leads to the dissociative mechanism [11].


$$\begin{array}{l} \text{Pt}+{\text{O}}_{2}\to {\text{PtO}}_{2}\;(\text{Single bonded adsorption}-\text{associative mechanism})\\ \text{2Pt}+{\text{O}}_{2}\to 2\text{PtO }(\text{Double bonded adsorption}-\text{dissociative mechanism}) \end{array}$$

The associative mechanism may also proceed in two basic routes, one leading to the formation of water by transfer of 4 electrons and the other leading to formation of hydrogen peroxide by transfer of 2 electrons. For higher performance in a fuel cell, the most desirable one is the 4 electron route. Hydrogen peroxide is also known to be corrosive to the fuel cell components. Practically, the reaction may be contributed by both pathways and the net number of transferred electrons per oxygen molecule can be any value between 2 and 4. For acidic conditions such as in PEM fuel cells, the oxygen is mainly reduced to water via a 4 electron pathway on Pt surface. There are many studies regarding ORR on catalysts other than Pt. Among those, Fe-N [12], Fe and Ni [13], Ag [14] and Pd, and Co-based catalysts [15,16] were studied for their ORR activity and mechanism. However, despite some considerable progress in these catalysts, platinum is still the most common choice of catalyst in fuel cells, especially for high-performance demanding applications. Instead of pure nonplatinum metals, better results were achieved by platinum-based multimetallic catalysts, where platinum is complemented with one or more transition metals [17–22]. Carbon-supported PtAgFe ternary catalysts have been shown to have mass activities comparable to that of commercial Pt/C catalyst [23].

The carbonaceous catalyst support may also have direct or indirect effects on the reaction mechanism [24,25]. In an indirect way, the carbon support may affect the physical properties (distribution, surface properties, etc.) of the metal catalyst or the diffusion rate of the oxygen and the reactant products. The carbon support may also directly affect the mechanism by itself getting involved in the ORR. However, studies concerning ORR mechanism on different types of carbon supports are rare. Ordered mesoporous carbons (OMCs) constitute a recently developed family of carbon materials, which are very promising as catalyst supports in fuel cells [26–28]. Compared with the carbon black, OMCs generally have higher surface areas and better defined (ordered) textures. Although the ordered structure may facilitate the mass transfer, the effect may be adversary in some cases depending on the texture (2d or 3d) and the pore size. Generally, a 3d interconnected pore structure with larger pores is advantageous both in terms of mass transfer and metal loading. Recently developed self-assembly techniques enable a more flexible synthesis route and produce carbons, which are better suited for fuel cell applications [29–32]. The self-assembly technique can be modified by the inclusion of silica species to obtain larger pores [33–35].

Although OMCs provide a good medium as a support, the metal loading technique is also very important for the distribution and activity of the catalyst particles. The widely used classical technique for catalyst loading is the wet impregnation-reduction in which the pores are filled with metallic salt precursors, and then the precursors are reduced to metal nanoparticles. This technique can be improved by modifying the surface with oxygen-containing functional groups before the impregnation process, in order to obtain a better metal distribution [36,37].

Despite extensive research on the kinetics and mechanism of ORR on carbon-black supported Pt catalysts, the number of studies regarding OMC supported Pt is very scarce. Here in this study, the ORR kinetics were investigated for Pt supported by OMC, which was synthesized by an organic-inorganic self assembly technique. Ternary PtAgFe catalysts supported by OMC were also examined for the first time. Catalyst particles were loaded by wet impregnation-reduction, both on surface modified and unmodified OMC. Activities of the catalysts and the kinetics of ORR were investigated in detail.

## 2. Experimental

### 2.1. OMC and OMC supported catalyst synthesis

Ordered mesoporous carbon (OMC) support was synthesized by an organic-inorganic self-assembly method, which was described elsewhere in detail [38]. In brief, Pluronic F127 (Aldrich) and resorcinol (Merck, %99) were dissolved in water-ethanol (2/3 v/v) solution. HCl was added to the solution as a polymerization catalyst. The acid concentration of the solution was 0.44 M. Then, formaldehyde (Aldrich, %37) and tetraethyl orthosilicate (Sigma–Aldrich, %98) were added so that the R/F and RF/F127 mass ratios would be 1/1 and 2/1, respectively. After the completion of polymerization, the polymer-TEOS composite was collected, dried, and carbonized in argon atmosphere with a controlled temperature up to 700 °C. Finally, the composite was etched by %15 HF solution for the removal of silica. Following this procedure, an OMC carbon with 8.1 nm mean pore size and 711 m^2^/g surface area was obtained [38].

**Figure 1:**
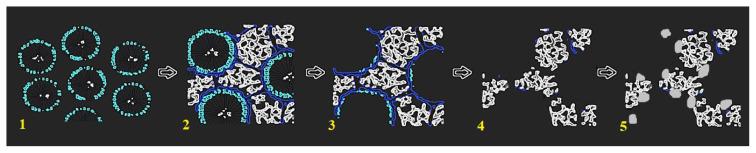
Schematic description of the synthesis process for Pt/OMC-W: 1) micelle formation, 2) polymerization around the micelle template, 3) carbonization and removal of template, 4) etching and removal of silica, 5) loading of Pt particles by wet impregnation.

Metal nanoparticles were loaded on the as-synthesized OMC by wet impregnation-reduction technique, as described earlier [39]. Metal precursors were used in appropriate concentrations to obtain metal loadings of 20% by weight. For loading of Pt, OMC was homogenized in chloroplatinic acid (H_2_PtCl_6_.6H_2_O) solution by ultrasonic blending for 30 min. Then, the chloroplatinic acid was reduced to metallic Pt by addition of sodium borohydride (NaBH_4_ %1 w/w) solution. The catalyst was filtered, dried, and heat treated at 500 °C. It was named Pt/OMC-W to denote wet impregnation-reduction. For another sample, the wet impregnation technique was modified by functionalization of the carbon surface before the loading. In this case, the OMC was refluxed in 2 M HNO_3_ for 2 h to create oxygen containing groups on the surface. The modified carbon was loaded with Pt by applying the same wet impregnation procedure, except that this sample was not heat treated to avoid spoilage of the surface groups. The resultant catalyst was named Pt/OMC-M to denote the modification. The loading of ternary PtAgFe catalysts on the modified and unmodified OMC basically followed the same route as with loading of pure Pt. The relative atomic ratios of the metals were adjusted to 6:1:1 for Pt, Ag and Fe, respectively. AgNO_3_ and Fe(NO_3_)_3_ were used as precursors for their corresponding metal. Actual metal contents determined by inductively coupled plasma mass spectrometry (ICP-MS) were given in Table 1. Commercial Pt/C catalyst (20% wt) purchased from Fuel Cell Store was used for comparison. A schematic description shows the basic steps in preparation of the support and catalyst loading (Figure 1).

### 2.2. Electrochemical characterization

Electrochemically active surface areas and oxygen reduction activities of the catalysts were measured by cyclic voltammetry (CV). Measurements were carried out with a AFCBP1 model Pine bipotentiostat in a three-electrode, single compartment reaction cell. Saturated calomel electrode (SCE), platinum wire, and glassy carbon disk covered by a thin film of catalyst were used as the reference, counter and working electrodes, respectively. For the preparation of the working electrode, 13 mg of catalyst was mixed with 200 μL nafion (5% w/w) solution and 250 μL deionized water to form a homogeneous slurry. A total of 10 μL of this slurry was dropped on the glassy carbon disk and allowed to dry in ambient air to form a thin catalyst film. 0.5 M H_2_SO_4_ solution was used as the electrolyte. The electrolyte was bubbled with pure nitrogen gas for 15 min to remove dissolved oxygen. Potential scanning was performed between −0.2 V and 1 V with 50 mV/s scan rate.

Rotating disk electrode voltammetry (RDE) was used to study the kinetics and the mechanism of the ORR. Measurements were performed in the same single cell and three electrode setup used in the CV measurements. In this case, the electrolyte solution was bubbled and saturated with high purity oxygen gas for 15 min. Linear potential scans were performed in the range of 0–1 V with a 10 mV/s scan rate at 5 different electrode rotation rates varying between 100–1600 rpm. Electrode rotation speed was controlled by a AFMSRCE model Pine rotor.

## 3. Results and discussion

### 3.1. Cyclic voltammetry measurements

**Figure 2:**
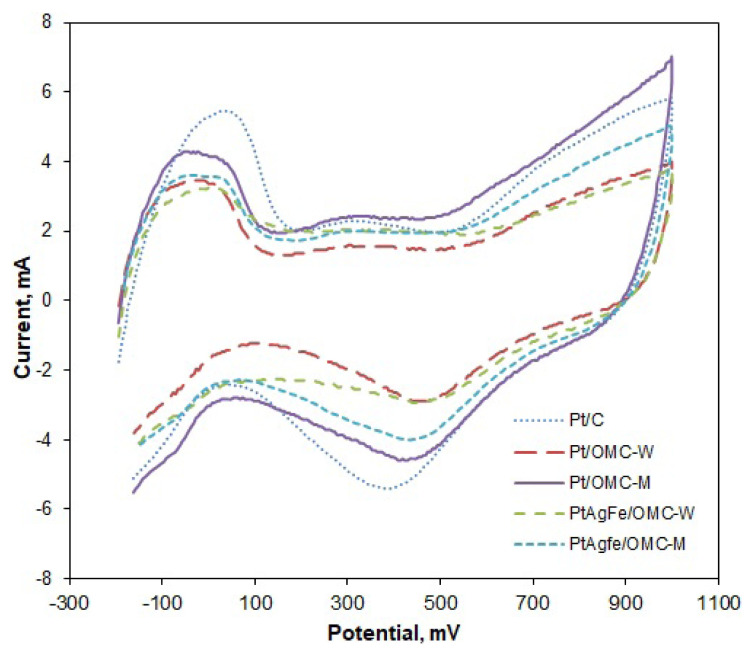
Cyclic voltammograms of OMC supported catalysts and the commercial Pt/C obtained in 0.5 M H_2_SO_4_ solution with a scan rate of 50 mV/s.

Cyclic voltammograms were obtained to measure the active surface areas and oxygen reduction activities of the catalysts (Figure 2). The broad peaks between −0.2 and 0.1 V at the anodic scan correspond to the desorption of hydrogen from the surface of Pt. This is followed by an increase in current at around 0.5 V due to formation of Pt oxides. Oxides are then reduced during the cathodic scan, between 0.4 and 0.5 V, and the cycle is completed by the adsorption of hydrogen onto the Pt surface at the low potential region. The area under the hydrogen desorption peak was used to calculate the electrochemically active surface areas (EAS), since this peak is associated with the charge transfer due to the desorption of a hydrogen monolayer. The almost steady current observed after the hydrogen desorption peak was due to the desorption of double-layer hydrogen. Therefore, the current under the extrapolated double layer region was subtracted from the total desorption peak area to find the actual charge due to the desorption of a monolayer. By integration, the EAS values were found as 1273 cm^2^/mgPt for Pt/C, 987 cm^2^/mgPt for Pt/OMC-M, and 865 cm^2^/mgPt for Pt/OMC-W. The EAS value of ternary PtAgFe/OMC-M catalyst was also measured as 899 cm^2^/mgPt (Table 2).

Since the highest EAS value was obtained from commercial Pt/C, it can be deduced that the wet impregnation of metals into the OMC support gave slightly larger-sized metal particles with lower surface areas. Among the OMC-supported catalysts, the surface modification led to higher EAS, indicating smaller-sized and better-distributed metal particles, while the Pt and PtAgFe catalysts on unmodified surfaces were fewer and larger. This is also consistent with the previously reported x-ray diffraction and transmission electron microscopy results [39].

The OMC-supported catalysts were found to possess considerably high EAS values, although still lower than Pt/C. Similar results were reported in the literature. For instance, one study found 1070 cm^2^/mg active area for Pt/OMC [27]. Hayashi reported 600 cm^2^/mg active surface area for mesoporous carbon-supported Pt catalyst [40]. Yoshii also reported an active surface area of 514 cm^2^/mg for Pt supported on Vulcan [41]. The EAS values of the surface modified OMC supported catalysts (Pt/OMC-M and PtAgFe/OMC-M) show a significant improvement with regard to the unmodified ones. This can be attributed to smaller particle size, or, in the case of PtAgFe catalysts, to lesser Pt content, in accordance with observations by Taylor [42].

Mass activities (MA) of the catalysts towards oxygen reduction were calculated from the ratio of the cathodic current at 0.65 V_SCE_ (equivalent to 0.891 V_RHE_) to the catalyst mass. According to this, Pt/OMC-M and the commercial Pt/C had the same mass activity with 39.1 mA/mgPt (Table 2). Despite having 25% less Pt content, PtAgFe/OMC-M showed an even higher mass activity of 44.8 mA/mgPt showing that the OR activity due to Pt is almost totally compensated by the Ag and Fe atoms. Another measure of activity is the specific activity (SA), which is defined as the ratio of the mass activity to the EAS of the catalyst. Pt/OMC-M catalyst had a higher specific activity compared to the Pt/C because the same mass activity is provided with a lower active surface area. This improvement in the specific activity possibly comes from the contribution of the active oxygen groups on the surface created by modification. This is supported by the lower specific activity of unmodified Pt/OMC-W catalyst. Among all catalysts, PtAgFe/OMC-M had the highest specific activity; thus, the most efficient use of platinum surface was obtained with this catalyst. The activities of OMC-supported catalysts of the present study are superior to that of many previously reported Pt-based catalysts. Hsueh reported a mass activity of 45.9 mA/mg, which is a close value to that obtained by PtAgFe/OMC-M [26]. Taylor measured the MA and SA of PtCo/C catalyst (20 wt%) as 9.9 mA/mg and 0.016 mA/cm^2^, respectively [42]. Another study reported a MA of 14.02 mA/mg and SA of 0.034 mA/cm^2^ by 18.96 wt% Pt_1_Pd_3_/OMC [43].

### 3.2. Rotating disk voltammetry measurements

**Figure 3:**
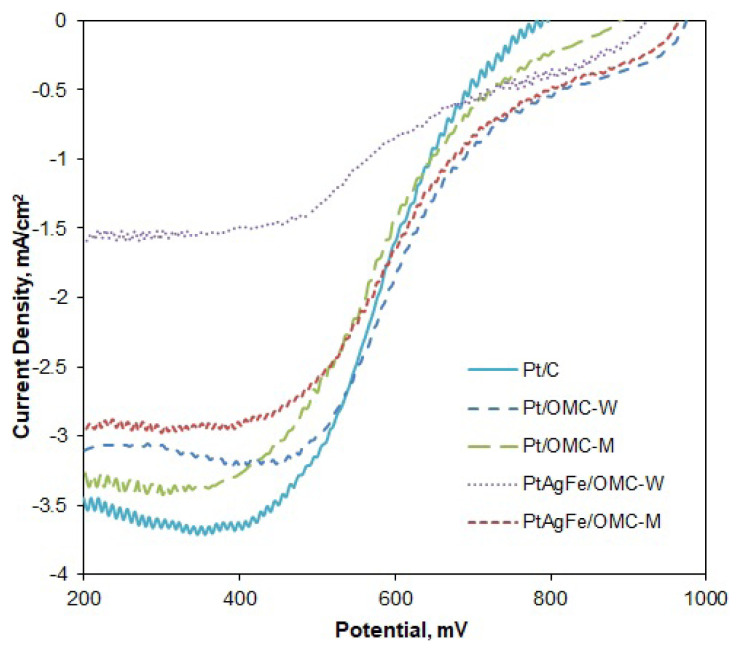
Comparison of the RDE voltammograms of the catalysts obtained in O_2_ saturated 0.5 M H_2_SO_4_ solution: ω = 1600 rpm, ν = 10 mV/s.

Oxygen reduction reaction (ORR) kinetics were investigated by rotating disk voltammetry. The potential was swept at different electrode rotation rates to find the relation between current, potential and the kinetic parameters such as oxygen concentration and the net number of transferred electrons per oxygen molecule. Limiting current densities (i_L_) are reached at low potential regions, where the diffusion rate of oxygen onto the catalyst surface is dominating the overall reaction rate. Figure 3 shows the RDE polarization curves obtained at 1600 rpm rotation rate for different catalysts. The diffusion regions are not perfectly flat, possibly due to microscopic level nonuniformities in the catalyst film thickness [44]. Except for PtAgFe/OMC-W, the limiting current values are close to each other and vary between 3 and 3.6 mA/cm^2^. Despite the considerable mass activity of PtAgFe/OMC-W, a weaker polarization curve indicates a kinetically controlled OR reaction in hydrodynamic conditions. The remaining OMC-supported catalysts and Pt/C had close i_L_ values, meaning that the overall oxygen diffusion rates were similar. The overall oxygen diffusion onto the catalyst surface may be affected by both the total available metallic surface and the bulk diffusion of oxygen in the porous medium. Since the cyclic voltammetry gave higher electroactive surface areas for Pt/C, the close limiting current values in OMC-supported catalysts are possibly due to faster bulk diffusion of oxygen within the ordered porous structure.

**Figure 4:**
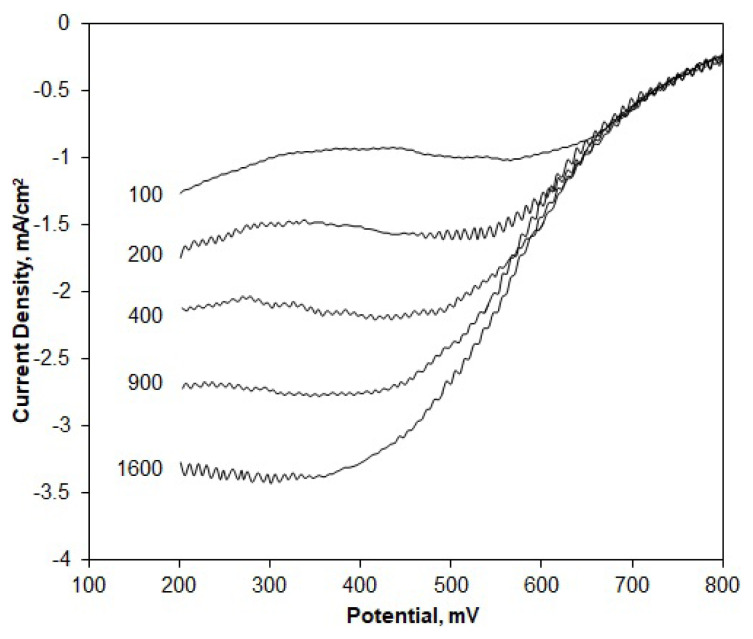
RDE voltammograms for the Pt/OMC-M catalyst obtained in O_2_ saturated 0.5 M H_2_SO_4_ solution at different electrode rotational rates: ω = 100–1600 rpm, ν = 10 mV/s.

RDE polarization curves were obtained for different rotation rates, ranging from 100 to 1600 rpm (Figure 4). The limiting current values clearly increase with the rotation rate due to faster oxygen diffusion. These curves were then used to construct the Koutecky–Levich plots, from which the net numbers of transferred electrons per oxygen atom were assessed. According to the Koutecky-Levich model, the resistance affecting the current at any potential point is the sum of the kinetic and diffusion resistances (Equation 1). The diffusion resistance is associated with the diffusion current, which is proportional to the B slope and the net number of transferred electrons (Equations 2 and 3).


(Eq. 1) $$\frac{1}{i}=\frac{1}{i_{k}}+\frac{1}{i_{d}}\;\;\;,\;\;\;i_{d}=B\omega^{-1/2}$$

(Eq. 2) $$\frac{1}{i}=\frac{1}{i_{k}}+\frac{1}{B\omega^{-1/2}}$$

(Eq. 3) $$B=0,62nF{\left[D_{O_{2}}\right]}^{2/3}\nu^{-1/6}C_{O_{2}}$$

**Figure 5:**
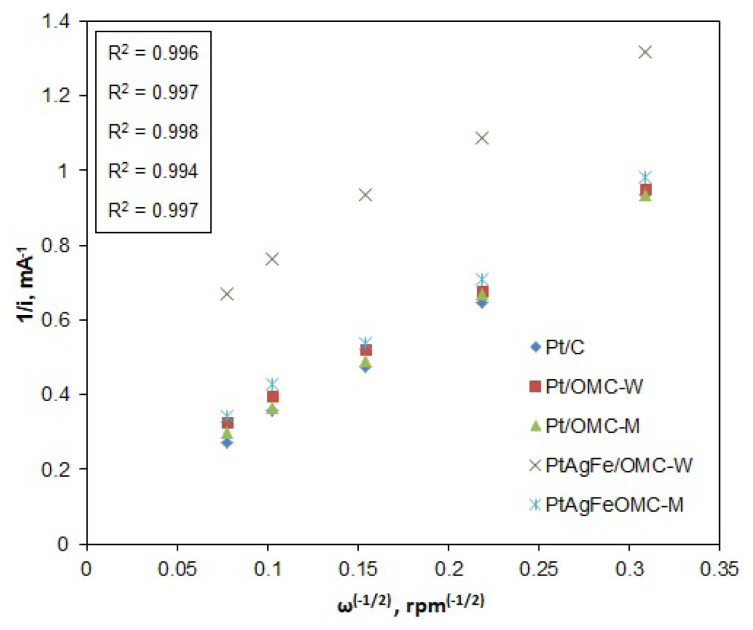
Comparison of the K-L plots for the catalysts, obtained from RDE current data corresponding to 0.3 V potential.

Here, n represents the net number of electrons transferred from the anode to the cathode per oxygen molecule, F is the Faraday constant (96500 C/mol), D_O2_ is the diffusivity of O_2_ in the electrolyte solution (1.9 × 10^−5^ cm^2^/s in 0.5 M H_2_SO_4_), ν is the kinematic viscosity of the solution (0.01 cm^2^/s), and C_O2_ is the bulk concentration of O_2_ (1.1 × 10^−6^ mol/cm^3^) in the electrolyte solution [45]. The n values were calculated by plotting 1/i versus 1/ω^−1/2^ data for any fixed potential from the polarization curves to obtain the so-called K-L plots and find the slope values (B). The K-L plots based on E = 0.3 V are given in Figure 5.

K-L plots of the catalysts are all linear and indicate first-order reaction rates with respect to oxygen. Plots are also very close except for PtAgFe/OMC-W, for which the polarization curves gave much lower current densities and significantly lower kinetic current values. This is also evidenced by the plot’s intersection with the y-axis, corresponding to 1/i_k_. This plot also has a lower R^2^ value and a slightly higher deviation from linearity, suggesting that the diffusion effects are less pronounced compared to the kinetic resistance. Indeed, PtAgFe/OMC-W had by far the smallest EAS value among OMC-supported catalysts, which caused the ORR to be mostly kinetic controlled. On the other hand, the K-L plots for all the catalysts gave similar B slopes, which means that the net number of transferred electrons (n) is similar. This suggests that the oxygen reduction mechanism is similar for all catalysts at the given potential. Values of n at 0.3 V were calculated as 3.31, 3.49, and 3.39 for Pt/C, Pt/OMC-W, and Pt/OMC-M, respectively. As for the ternary catalysts, PtAgFe/OMC-W and PtAgFe/OMC-M had n values of 3.38 and 3.45, respectively. The difference of 0.14 between the lowest and highest values of n, corresponds to 7% difference in peroxide formation percentage.

**Figure 6:**
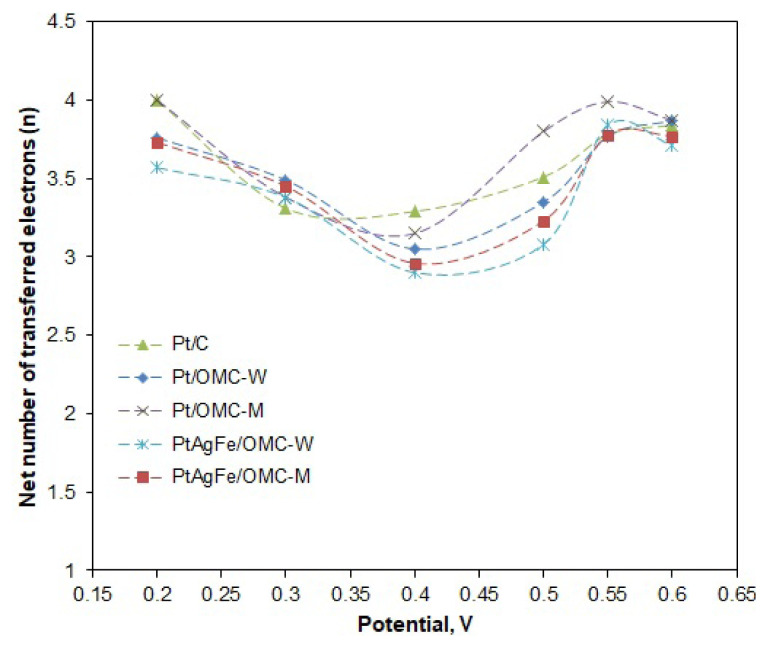
Variation of the net number of electrons transferred from anode to cathode (*n*) with the potential.

**Figure 7:**
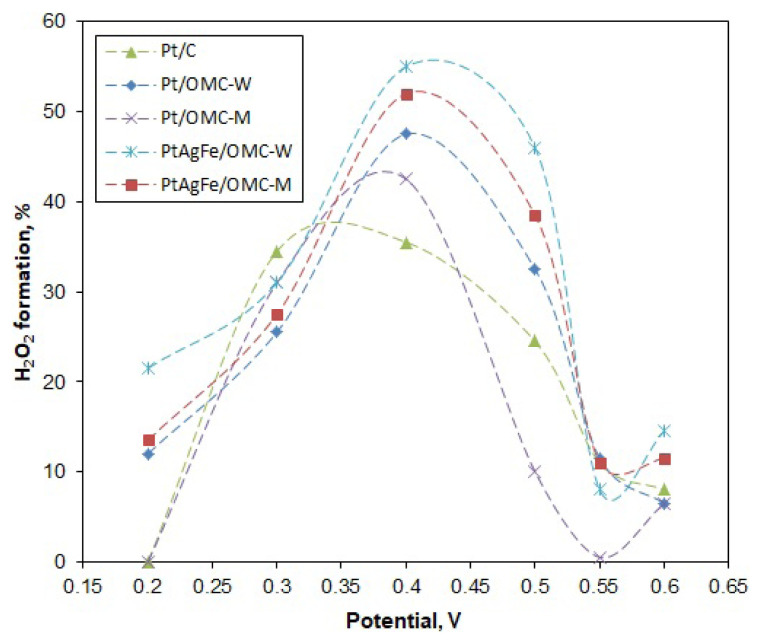
Variation of the peroxide formation percentage with the potential.

Likewise, n values were calculated for different potentials to see the variation of n with the potential (Figure 6). The potential range was taken between 0.2 and 0.6 V, since the deviations from linearity of the K-L line become increasingly higher over 0.6 V (mixed diffusion region). The n values were close and varied between 3 and 4 throughout much of the potential region. The 4-electron pathway was seen to be more dominant at low potentials, with Pt/C and Pt/OMC-M having n values of 4 at 0.2 V. The n values were used to calculate the hydrogen peroxide formation percentages. As seen in Figure 7, the peroxide formation is at the lowest for low potentials and increases with the potential, peaking around 0.4 V and then decreasing again. A similar trend was observed by Cheon using ordered mesoporous porphyrinic carbon-supported metallic catalysts [46]. For much of the potential region, the Pt/OMC-M catalyst gave a lesser peroxide formation, particularly over 0.45 V.

These results are in agreement with those previously reported for different catalysts and supports. For instance, the n value for clay-supported Pt was reported to vary between 3.2 and 3.8 [47]. ORR kinetics over mesoporous carbons, however, is a rather scarcely studied subject. Liu reported that the n values were between 3.5 and 3.6 in best case for N-modified OMC supported Pt catalysts [48]. Viva reported n values of 3 and 3.2 for mesoporous carbon supported Pt and Pt/C respectively, deducing that the OR mechanism is similar for those two catalysts [49]. Our results showed that both the Pt and PtAgFe catalysts on OMC had n values closer to 4, and oxygen was predominantly reduced to water.

### 3.3. ORR mechanism on Pt/OMC and PtAgFe/OMC catalysts

To investigate the ORR mechanism, Tafel plots were constructed for the kinetic and mixed current regions (0.6–0.9 V) of the RDE voltammograms. Polarization curves obtained at ω = 1600 rpm, where the diffusion resistance is at minimum, were taken as the basis. The kinetic current values (i_k_) were calculated from Eq. 1. According to the empirical Tafel model, the overpotential (*ΔE*) at mixed and diffusion current regions is proportional to the logarithm of kinetic current; thus, the cathodic current is inversely proportional to the logarithm of kinetic current (Eq. 4).


(Eq. 4) $$\Delta E=a+blog(i_{k}),\;\;\;\Delta E=E_{0}-E_{c}$$

**Figure 8:**
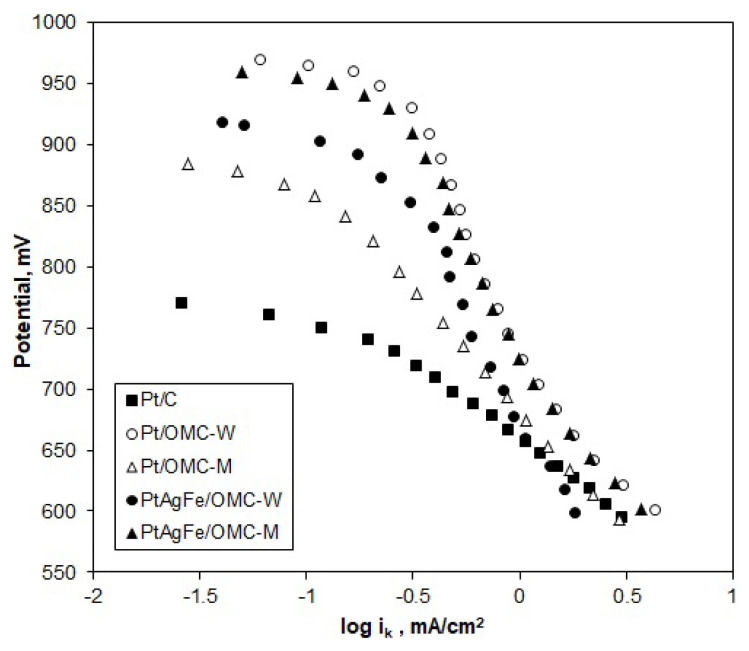
Tafel plots of the catalysts obtained from the kinetic and mixed current regions of RDE voltammograms in O_2_ saturated H_2_SO_4_; ω = 1600 rpm, ν = 10 mV/s.

The slope of the E-logi_k_ curves gives the Tafel slope b, which is characteristic of the reaction mechanism. At high potentials, the surface composition of the electrode surface is a mixture of Pt and PtO, while, at lower potentials, Pt is mostly in metallic form. Therefore, variations in Tafel slope indicate a change in the mechanism and surface composition of the catalysts with the potential. Generally, the ORR is assumed to follow the 4-electron dissociative mechanism at the low-current density range and the 2-electron associative mechanism at the high-current density range [50,51]. In Figure 8, the Tafel plots of the catalysts are in good agreement with the two-region theory, which assumes that different slopes indicate changing preferences for the associative mechanism or dissociative mechanism.

The Tafel slopes of Pt/C were −50 mV/dec and −120 mV/dec for b_1_ and b_2_, respectively, consistent with the literature. Low Tafel slopes around −60 mV/dec indicate a dissociative mechanism with a pseudo 2-electron reaction as the rate-determining step (rds) [24]. Pt/OMC-M gave slopes of b_1_ = −55 mV/dec and b_2_ = −165 mV/dec, indicating a similar ORR mechanism with Pt/C at high potentials. However, since the b_2_ region extended to a greater potential range, it can be said that the ORR on Pt/OMC-M predominantly followed the associative mechanism. In the case of Pt/OMC-W, the first Tafel slope starts at around −50 mV/dec, while the second and more dominant curve, spanning to a greater potential range has a value of −260 mV/dec. Thus, the associative mechanism is the dominant mechanism as in Pt/OMC-M. In this mechanism, the first electron transfer to the oxygen molecule is the rate-determining step, which leaves a weaker O bond to be easily broken [52]. The significantly low Tafel slope (b_2_ = −260 mV/dec) indicated a low oxygen concentration on the Pt surface, so the ORR on this catalyst can be assumed to be mixed activation-diffusion controlled. The PtAgFe catalysts also had very similar Tafel slopes with the Pt/OMC-W, both at low and high potentials, showing that the Ag and Fe atoms did not alter the reaction mechanism in a significant way. In fact, the predominant associative mechanism and the relatively higher peroxide formation rates from Figure 7 suggest that neighboring Ag and Fe atoms may facilitate single-bond adsorption on Pt.

Considering the Tafel slopes, the ORR on OMC supported catalysts was seen to follow the dissociative mechanism at high potentials (850 mV and above) and the associative mechanism at the mid-potential region. In the dissociative mechanism, the O-O bond is broken just after the adsorption of oxygen molecules onto the Pt surface. As for the associative mechanism, O-O bond is kept intact after the adsorption process. The 2-electron pathway (peroxide route) is only possible in the case of associative mechanism, since it requires an intact O-O bond after adsorption. The fact that the net number of transferred electrons obtained from K-L plots is close to 4 shows that the water formation route is predominant even when an associative mechanism is followed. In general, results show that the reaction mechanisms are similar for all catalysts and follow a 4 electron transfer route at high potentials, while the changing Tafel slopes at lower potentials indicate shiftings in favor of associative mechanism.

## 4. Conclusions

Kinetics of ORR were studied on OMC-supported Pt and PtAgFe catalysts. Cyclic voltammetry measurements showed that most of the OMC-supported catalysts have EAS values comparable with that of commercial Pt/C, with the highest EAS measured for Pt/OMC-M with 987 cm^2^/mg_Pt_. Catalysts with modified supports had significantly higher EAS values compared with those with unmodified supports. In particular, the high mass activity of PtAgFe/OMC-M catalyst with 44.8 mA/mg_Pt_ showed an enhancement in the the oxygen reduction efficiency of Pt in the presence of Ag and Fe. According to RDE measurements, the limiting current densities were as high as 3.4 mA/cm^2^ for OMC supported catalysts. The linearity of Koutecky–Levich plots indicated first order reaction rates with respect to oxygen for all catalysts. The net number of transferred electrons varied with potential in the range of 3 and 4, indicating a high preference for the water formation route. The PtAgFe catalysts had slightly higher peroxide formation rates. Tafel plots showed that OMC-supported catalysts had similar oxygen reduction mechanisms with the carbon black supported commercial Pt/C, with the difference that the associative mechanism was pronounced at a wider potential range. Higher peroxide rates in PtAgFe catalysts also indicated a preference for the single-bond adsorption associative mechanism. The modified OMC supported catalysts showed greater OR activities in general, however, the enhancement was most probably due to particle and surface effects rather than mechanistic.

## Figures and Tables

**Table 1:** Actual metal contents of the catalysts determined by ICP-MS.

Catalyst	Metal content, % w/w		
	Pt	Ag	Fe
Pt/OMC-M [39]	18.4	-	-
Pt/OMC-W [39]	16	-	-
PtAgFe/OMC-M	13.8	2.2	2.3
PtAgFe/OMC-W	12.6	2.4	2.2

**Table 2:** Electrochemical properties of the catalysts (Q_h_; Charge associated with the monolayer desorption of hydrogen, EAS; electrochemically active surface area, MA; mass activity, SA; specific activity.

Catalyst	Q_h_, mC	EAS, cm^2^/mg_Pt_	MA, mA/mg_Pt_	SA, mA/cm^2^
Pt/C^®^	14.97	1273	39.1	0.031
Pt/OMC-M	11.61	987	39.1	0.040
Pt/OMC-W	10.18	865	23.6	0.027
PtAgFe/OMC-W	5.28	598	37.2	0.062
PtAgFe/OMC-M	7.93	899	44.8	0.050
